# Genomic and phenotypic characterization of staphylococci isolated from the skin of non-human primates

**DOI:** 10.1099/mic.0.001546

**Published:** 2025-03-25

**Authors:** Caitlin Wildsmith, Simon Barratt, Frances Kerridge, Jonathan Thomas, David Negus

**Affiliations:** 1 Department of Biosciences, Nottingham Trent University, Nottingham, UK; 2 Department of Animal Science, Myerscough University Centre, Preston, UK

**Keywords:** antimicrobial resistance (AMR), biofilm, coagulase-negative *Staphylococcus*, prophage, virulence, whole-genome sequencing

## Abstract

The growth of wildlife tourism coupled with continued deforestation has resulted in increased contact between non-human primates (NHPs) and humans. Such events may promote the transmission of potentially pathogenic bacteria such as *Staphylococcus* spp. However, the presence and associated virulence of staphylococci associated with NHPs remain poorly characterized. To help address this, we isolated staphylococci from the skin of four NHP species housed at a UK zoo and characterized their antimicrobial resistance, virulence factors and prophage. We recovered 82 isolates from mannitol salt agar, of which 28 were tentatively confirmed as staphylococci by 16S rRNA gene sequencing. Fourteen isolates were determined to be unique, based on differences in their 16S rRNA gene sequences and origins of isolation. Whole-genome sequencing of the 14 isolates and subsequent genomic analysis identified 5 species, belonging to the genus *Staphylococcus* (*Staphylococcus aureus*, *Staphylococcus epidermidis*, *Staphylococcus pasteuri*, *Staphylococcus saprophyticus* and *Staphylococcus warneri*). Bioinformatic prediction of antimicrobial resistance genes identified a total of 85 resistance determinants across all 14 isolates, potentially rendering them resistant to a range of antibiotic classes. However, phenotypic testing revealed only a single case of clinical resistance. Isolates belonging to the species *S. pasteuri* were identified as the most proficient biofilm formers. Potentially complete prophages were identified in 11 of the sequenced isolates. Prophage JCT0104_p1, identified within the genome of *S. aureus* JCT0104, was found to encode the virulence factor staphylokinase, which is associated with pathogenesis in humans. Our findings contribute to the limited knowledge of the diversity and characteristics of staphylococci residing on the skin of captive NHPs.

## Data Availability

The sequences for genomes described herein have been deposited with the NCBI under BioProject accession PRJNA1113975, and individual assembly accessions can be found in Table 1.

Supplementary data and material associated with this article are available from figshare at https://figshare.com/projects/Genomic_and_phenotypic_characterisation_of_staphylococci_isolated_from_the_skin_of_non-human_primates/204126

## Introduction

The *Staphylococcaceae* family comprises a diverse group of non-motile, non-spore-forming Gram-positive cocci, with the genus *Staphylococcus* containing the greatest abundance of species. Species belonging to the genus *Staphylococcus* are typically divided into coagulase-positive staphylococci (CoPS) or coagulase-negative staphylococci (CoNS), based on their ability to produce the blood-clotting enzyme coagulase. CoPS species, such as *Staphylococcus aureus*, are renowned opportunistic pathogens known for their extensive production of toxins and acquired antibiotic resistance mechanisms, which contribute to potentially lethal skin, lung and blood infections [1,3]. Conversely, CoNS species produce fewer virulence factors and have traditionally been considered commensal members of the skin microbiota of humans and a diverse range of animals [4,5]. Nevertheless, recent studies have shown that CoNS species can act as opportunistic pathogens capable of causing infections in immunocompromised individuals when the skin barrier has been compromised [5,7]. These infections can manifest in a variety of clinical forms; for instance, *Staphylococcus epidermidis* is currently the most common cause of central line-associated bloodstream infections in hospitalized patients, while *Staphylococcus chromogenes*, *S. epidermidis*, *Staphylococcus devriesei*, *Staphylococcus xylosus* and *Staphylococcus haemolyticus* are causative agents of clinical and subclinical bovine mastitis [3,7, 8]. Such infections are becoming increasingly difficult to treat due to biofilm formation and the acquisition of antibiotic resistance genes [4,9]. Given the potential disease risk these opportunistic pathogens pose, extensive research efforts have been dedicated to investigating the abundance and virulence of *Staphylococcus* spp. residing on the skin of humans, companion animals and livestock. However, limited attention has been given to the prevalence of staphylococci associated with wild animals, one justification being the challenges of obtaining samples without negatively impacting the sampler, the animal or the animal’s natural environment [10].

The potential for transmission of bacteria between non-human primates (NHPs) and humans has been highlighted by the isolation of human-specific methicillin-susceptible *S. aureus* (MSSA) lineages from green monkeys (*Chlorocebus sabaeu*s) in The Gambia [11]. It was believed that this transfer occurred due to humans directly hand-feeding the monkeys. Additional studies have also identified the transfer of staphylococci between humans and NHPs [12,13], and such transmission events are likely to become more frequent as we continue to encroach on NHP habitats.

Previous reports documenting the recovery of staphylococci from NHP skin have focused primarily on the carriage of methicillin-resistant *S. aureus* (MRSA) [11,14,16]. However, relatively little is known about the prevalence, diversity and pathogenicity of other *Staphylococcus* spp. associated with NHP skin. Therefore, we collected skin swabs from captive lemurs, spider monkeys, marmosets and tamarins with the aim of isolating and genome sequencing a diverse range of staphylococci. Here, we report the characterization of these isolates with regard to their species distribution, genomes, antibiotic resistance profiles, virulence genes and prophages.

## Methods

### Sample processing and isolation of *Staphylococcus* spp.

Dry skin swabs were collected from the palms of 16 NHP individuals housed in Reaseheath Zoo in 2016. NHPs included ring-tailed lemurs (*n*=4), black lemurs (*n*=2), common marmosets (*n*=4), cotton-top tamarins (*n*=3) and spider monkeys (*n*=3). Within 12 h of sampling, swabs were transferred to tryptone soy agar plates (Oxoid) and incubated overnight at 37 °C. For selective isolation of staphylococci, all morphologically distinct colonies were subbed onto mannitol salt agar (MSA; Oxoid) and incubated overnight at 37 °C.

### Genomic DNA extraction

Genomic bacterial DNA was extracted from overnight liquid cultures grown in 10 ml brain heart infusion broth (Oxoid) at 37 °C with orbital shaking (150 r.p.m.). One millilitre of liquid culture was centrifuged (6,000 ***g***, 5 min), and the cell pellet was resuspended in 200 µl of enzymatic lysis buffer (20 mM Tris-Cl, pH 8.0, 2 mM sodium EDTA, 1.2% Triton X-100, 20 mg ml^−1^ lysozyme and 25 µg ml^−1^ lysostaphin) and incubated at 37 °C for 1 h. DNA was extracted using a QIAGEN DNeasy blood and tissue kit (QIAGEN) following the Gram-positive extraction protocol. DNA was eluted in 50 µl of pre-warmed elution buffer (QIAGEN). The DNA concentration was quantified using an Invitrogen Qubit 4 fluorometer with the HS assay kit (Thermo Fisher Scientific, UK). DNA purity was checked using a NanoDrop 2000.

### PCR and sequencing of 16S rRNA gene products

The amplification of the 16S rRNA gene was performed using the 27f forward (5′-AGAGTTTGATCCTGGCTCAG-3′) and 1492r reverse (5′-GGTTACCTTGTTACGACTT-3′) 16S rRNA primers (Macrogen) diluted to 10 µM in DNase-free H_2_O (Invitrogen). PCR reactions contained 1 µl of genomic DNA, 25 µl MangoMix (Meridian Bioscience), forward and reverse primers (0.5 µM final concentration) and 23 µl DNase-free H_2_O. Thermocycler conditions were as follows: initial denaturation, 94 °C for 5 min; 30 cycles, 94 °C for 1 min, 55 °C for 1 min and 72 °C for 2 min; and final extension, 72 °C for 5 min. Gel electrophoresis was performed to confirm the presence of a single band of the expected size (1,445 nt) against a GeneRuler 1 kb ladder (Thermo Scientific). PCR products were purified using the Gene JET PCR Purification Kit (Thermo Fisher Scientific). The concentration of purified DNA was quantified using an Invitrogen Qubit 4 fluorometer, and the purity was checked using a NanoDrop 2000. The concentration of DNA fragments was adjusted to 10 ng µl^−1^ prior to Sanger sequencing by Source BioScience using the forward primer.

Returned 16S rRNA gene sequences were trimmed in Geneious Prime v11.0.14.1 to remove ambiguous bases. Trimmed sequences were queried against the EzBioCloud 16S database web server v20210707 to determine tentative taxonomy.

### Whole-genome sequencing

Whole-genome sequencing (WGS) data were generated using our in-house Illumina MiSeq platform. Genomic DNA was adjusted to a concentration of 0.2 ng µl^−1^, and following the manufacturer’s instructions, the Nextera XT DNA library preparation kit (Illumina) was used to produce indexed libraries containing fragments of ~500 bp. DNA libraries were pooled, denatured and adjusted to 8 pM, following Illumina’s recommended procedures, before 2×250 paired-end sequencing using the MiSeq Reagent Kit v2 (Illumina).

### Genome assembly

Sequencing reads were assembled using default parameters for all software except where otherwise specified. Adapter sequences were removed from raw Illumina reads using Trimmomatic v0.39 [17], and duplicate reads were removed using clumpify.sh v38.34 from the BBTools package. Genomes were assembled *de novo* using SPAdes v3.14, with all contigs below 500 nt removed [18]. Genome assembly quality was determined with CheckM v1.2.3 [19].

### Genomic analyses

To confirm the bacterial taxonomy of assembled draft genomes, ribosomal MLST (rMLST) was carried out using the PubMLST web server (accessed: 09 May 2024) [20]. Sequence types (STs) of *S. epidermidis* and *S. aureus* were determined using PubMLST species-specific typing databases (accessed: 09 May 2024).

Prophages encoded within genomes were predicted using PHASTEST v3 [21]. Prophages were annotated using pharokka v1.3.2 [22], and the closest known phage relatives based on genome-wide sequence similarity were identified using proteomic trees generated with ViPTree v4 [23]. Fasta files for the closest known phage relatives were retrieved from the NCBI and annotated using pharokka v1.3.2. Homologous protein group comparisons between prophages and their closest known phage relatives were visualized using LoVis4u v0.1.1 [24]. To aid genomic comparisons, prophage genomes were re-orientated to begin with the integrase gene. In cases where potentially truncated prophages lacked the integrase gene, alignments were re-orientated to begin with the first commonly named gene, as identified through pairwise alignment.

Bacterial genomes and predicted intact prophages were screened for antimicrobial resistance (AMR) genes using the Resistance Gene Identifier v6.0.3 of the Comprehensive Antibiotic Resistance Database (CARD) v3.3.0 [25] using selection criteria ‘perfect and strict hits only’ and for virulence genes using VFanalyzer of the Virulence Finder database (accessed: 09 May 2024) [26] with *Staphylococcus* selected as the reference genus.

Average nt identities (ANIs) were determined using FastANI v1.3 [27]. Type strains and their assembly accession numbers used in this study are as follows: *Staphylococcus warneri* DSM 20316 (GCA_029023845.1), *Staphylococcus pasteuri* DSM 10656 (GCA_003970495.1), *S. aureus* ATCC 12600 (GCA_006094915.1), *Staphylococcus saprophyticus* NCTC 7292 (GCA_000010125.1) and *S. epidermidis* ATCC 14990 (GCA_006094375.1).

### Phylogenetic analysis

The NCBI blast command-line package tblastn was used to identify seven *Staphylococcus* orthologous group genes within the genomes, namely, *mutS*, *recD2*, *pepA*, *yheS*, *pheT*, *pbp3* and *rpoC*, as proposed by Graña-Miraglia *et al*. [28,29]. Identified genes with ≥90% coverage to the reference gene were extracted from genome fasta files using the Perl package fasta_subgrep.pl, and genes were concatenated in the order outlined above. Geneious Prime and the in-built multiple sequence aligner muscle v3.8.425 [30] feature was used to generate a multiple alignment for the concatenated seven genes, which was used to create a maximum likelihood tree using the PHYML v3.3.21080621 plugin in Geneious Prime [31] and the HKY85 model [32]. To evaluate the degree of statistical support for the nodes on the tree, their per cent recovery was examined in 1,000 resampled trees using the non-parametric bootstrap test. The phylogenetic tree was uploaded to the iTOL web server v6.6 for annotation and visualization [33].

### Phenotypic confirmation testing

#### Antibiotic disc diffusion assays

Antibiotic resistance profiles were determined phenotypically using disc diffusion assays against a panel of seven antibiotics: tetracycline (30 µg), cefoxitin (30 µg), gentamycin (10 µg), erythromycin (15 µg), sulfamethoxazole and trimethoprim (25 µg), rifampicin (5 µg) and clindamycin (2 µg) (Oxoid). Disc diffusion assays were conducted in accordance with the European Committee on Antimicrobial Susceptibility Testing (EUCAST) guidelines, and zones of inhibition were interpreted using the EUCAST clinical breakpoints v13.0. *S. aureus* ATCC 29213 was used as the reference strain for quality control purposes, and * S. aureus* NCTC 1249 was used as a resistant positive control.

#### Biofilm assays and interpretation

Biofilm assays were performed as described previously [34,36]. In brief, overnight bacterial cultures in tryptone soy broth (TSB) were adjusted in fresh medium to an OD (OD_600_ nm) of 0.1. Then, 100 µl of adjusted culture or sterile TSB (negative control) was pipetted in 4 wells of a 96-well plate, followed by incubation for 24 h at 37 °C under static conditions. The turbid broth was carefully tipped away, and the wells were washed with 200 µl of PBS pH 7.4 (Oxoid) three times to remove planktonic cells. Adhered cells were heat fixed at 60 °C for 1 h before staining with 150 µl 0.1% crystal violet (w/v) (LabM) for 20 min. The stain was carefully tipped away, and the wells were washed with water three times to remove unbound dye. The plates were inverted to dry, and the biofilm was solubilized with the addition of 150 µl 33% (v/v) glacial acetic acid (100063, EMSURE). Absorbance readings at OD_540_ nm were recorded using a BioTek Cytation imaging reader. Biological (*n*=3) and technical (*n*=4) replicates were completed. *S. epidermidis* ATCC 35983, a clinical isolate and known biofilm former [37], was included as a positive control. The mean of each isolate’s OD quadruplicate readings (OD_i_) was calculated and compared with the control cut-off OD (OD_C_), which was defined as three sd above the mean of the negative control (3sd+mean). The amount of biofilm formed was scored as non-adherent (OD_i_≤OD_C_), weakly adherent (OD_C_<OD_i_≤2 OD_C_), moderately adherent (2 OD_C_<OD_i_≤4 OD_C_) or strongly adherent (4 OD_C_<OD_i_).

## Results

### 16S rRNA gene sequencing analysis

We recovered 82 morphologically unique colonies on MSA from 12 of the 16 NHP individual swabs. The remaining four swabs (cotton-top tamarins=3 and common marmoset=1) did not yield bacterial growth following enrichment in TSB. Sanger sequencing and analysis of trimmed 16S rRNA gene sequences tentatively identified 28 isolates as members of the genus *Staphylococcus*. The remaining 54 isolates were members of the genera, *Enterococcus*, *Enterobacter*, *Bacillus*, *Leclercia*, *Pantoea* and *Kosakonia*, and were not taken any further in our investigations. The 16S rRNA gene sequences of all 82 strains have been made available on Figshare as a multi-fasta file; their lab ID, source of isolation and tentative taxonomy supported by sequence similarity score are detailed in Table S1 (available in the online Supplementary Material). From the remaining 28 *Staphylococcus* isolates, 14 were unique, based on differences in their 16S rRNA gene sequences and origins of isolation; these 14 were further investigated by Illumina WGS.

### WGS analysis

Fourteen high-quality draft genomes were assembled with completeness scores of >97% and contamination scores of <2% as determined by CheckM. Accession numbers and assembly statistics can be found in Table 1 and Table S2.

**Table 1.** NHP staphylococcal whole-genome sequences. Type strains used: *S. warneri* DSM20316, *S. pasteuri* DSM10656, *S. aureus* ATCC12600, *S. saprophyticus* NCTC7292, *S. epidermidis* ATCC14990

Lab ID	Accession	NHP common name	Individual swab	rMLST ID, % support	ANI (%) against type strain
JCT0097	JBFQYQ000000000.1	Spider monkey	1	*S. pasteuri* (100%)	98.86
JCT0099	JBFQYP000000000.1	Spider monkey	2	*S. pasteuri* (100%)	98.88
JCT0104	JBDQZG000000000.2	Spider monkey	2	*S. aureus* (100%)	97.74
JCT0114	JBFQYO000000000.1	Spider monkey	3	*S. saprophyticus* (100%)	99.43
JCT0126	JBFQYN000000000.1	Common marmoset	7	*S. epidermidis* (100%)	99.44
JCT0140	JBFQYM000000000.1	Black lemur	12	*S. warneri* (45%)	95.76
JCT0146	JBDQZF000000000.2	Black lemur	12	*S. warneri* (45%)	95.73
JCT0155	JBDQZE000000000.2	Ring-tailed lemur	14	*S. warneri* (45%)	95.87
JCT0158	JBDQZD000000000.2	Ring-tailed lemur	14	*S. warneri* (45%)	95.90
JCT0159	JBDQZC000000000.2	Ring-tailed lemur	14	*S. pasteuri* (98%)	98.81
JCT0167	JBDQZB000000000.2	Ring-tailed lemur	15	*S. pasteuri* (98%)	98.73
JCT0175	JBDQZA000000000.2	Black lemur	17	*S. warneri* (45%)	95.80
JCT0178	JBDQYZ000000000.2	Black lemur	17	*S. pasteuri* (89%)	98.90
JCT0179	JBDQYY000000000.2	Black lemur	17	*S. warneri* (45%)	95.81

Isolate taxonomy was initially determined by rMLST and confirmed by ANI (≥95%) against the respective species type strain (Table 1). Both CoPS and CoNS species were identified; CoNS species included *S. pasteuri*, *S. warneri*, *S. epidermidis* and * S. saprophyticus,* while the single CoPS species was *S. aureus*.

### Genetic diversity of isolates

**Fig. 1.**
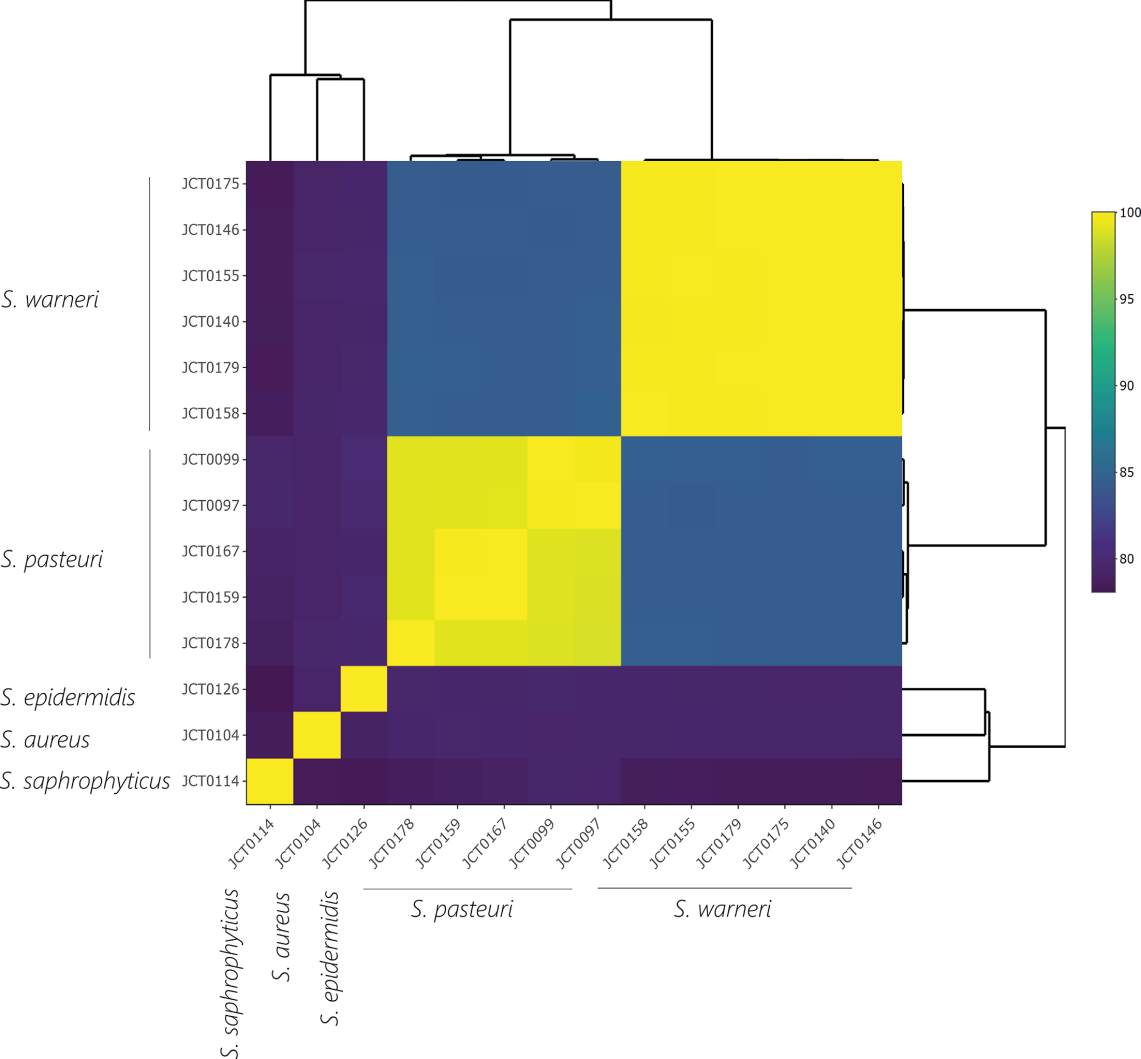
Heatmap and hierarchical clustering of *Staphylococcus* whole-genome sequences by FastANI values using an all-vs-all comparison.

**Fig. 2.**
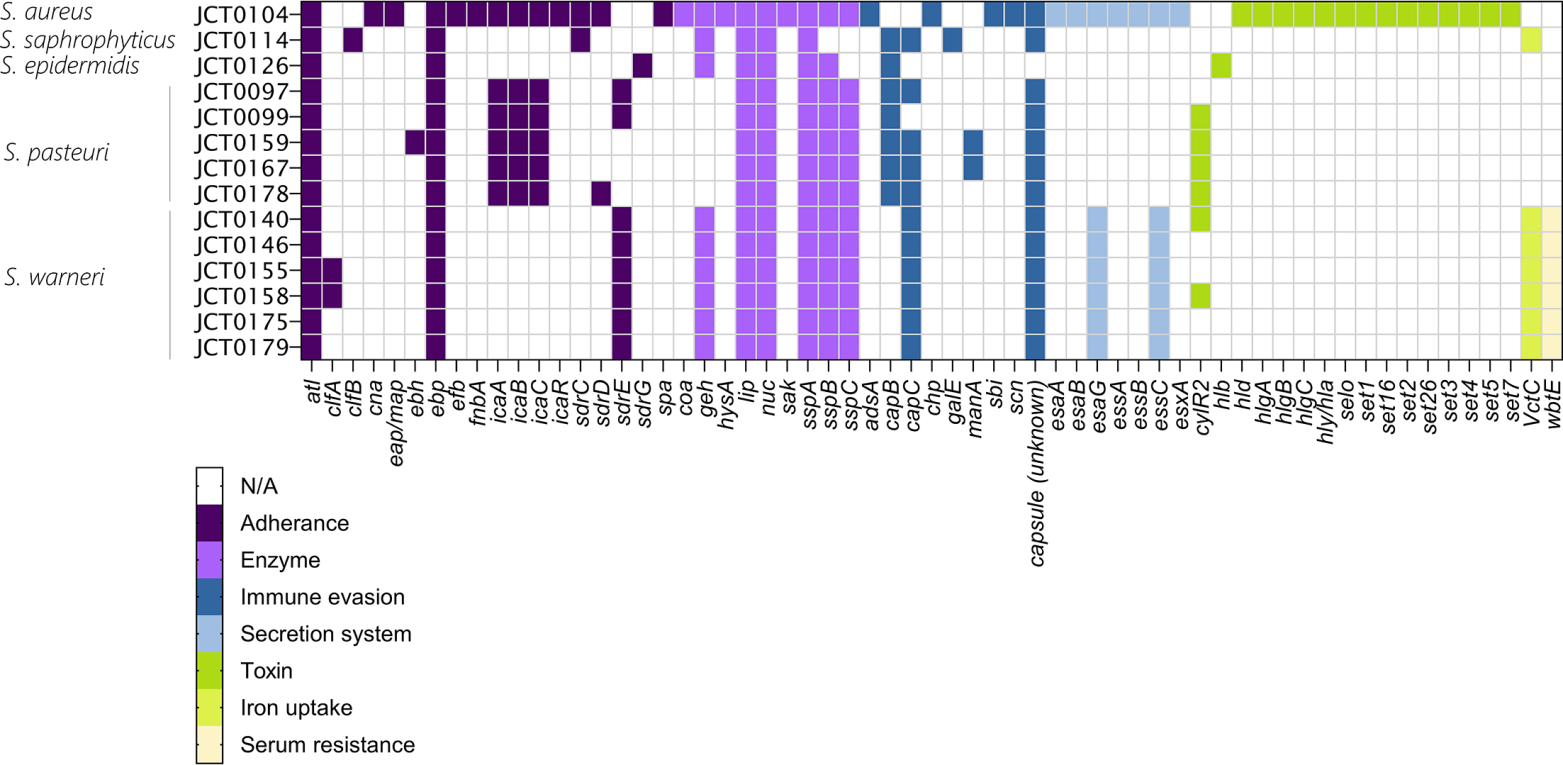
Heatmap showing the distribution of virulence genes across staphylococcal strains colour coded by virulence mechanism.

The genetic diversity of the 14 *Staphylococcus* isolates was determined using FastANI (Fig. 1). All strains shared an ANI score of between 78% and 99%. Based on 16S rRNA gene similarity, JCT0140, JCT0158 and JCT0175 were initially identified as members of an uncharacterized species, designated *Staphylococcus* CP022881_s. However, subsequent WGS and FastANI analysis revealed that these isolates share ≥95% ANI with *S. warneri* strain DSM20316^T^, leading to their reclassification as *S. warneri*. All six strains of *S. warneri* shared an ANI score of 99% when compared to each other (Fig. 1). Despite high levels of sequence similarity and some of the *S. warneri* strains being isolated from the same NHP individual, they do not appear to be clonal. For example, although JCT0155 and JCT0158 were both isolated from ring-tailed lemur individual 14 and share 99% ANI, there are differences in the predicted virulence genes they possess (Fig. 2). Similarly, JCT0140 and JCT0146 were both isolated from black lemur individual 12, but the number of predicted virulence genes differs between the two strains (Fig. 2).

The five strains of *S. pasteuri* were isolated from different individual spider monkeys or ring-tailed lemurs. *S. pasteuri* strain JCT0097 (isolated from spider monkey individual 1) and JCT0099 (isolated from spider monkey individual 2) shared an ANI of 99%. Similarly, *S. pasteuri* JCT0159 (isolated from ring-tailed lemur individual 14) and JCT0167 (isolated from ring-tailed lemur individual 15) shared an ANI of 99%. However, the ANI score dropped to 98% similarity when *S. pasteuri* strains of differing NHP species were compared.

### Sequence typing

Of the species isolated in this study, species-specific MLST schemes are currently only available for strains of *S. aureus* [38] and *S. epidermidis* [39]. *S. epidermidis* (JCT0126) belonged to ST621, a member of the putatively hospital-adapted genetic cluster (GC) 5 [40], while *S. aureus* (JCT0104) belonged to ST39 and clonal complex (CC) 30.

### Phylogenetic analysis

Phylogenetic analysis was based on seven *Staphylococcus* orthologous group genes identified as markers for species phylogeny in the genus *Staphylococcus* [28]. The maximum likelihood tree demonstrated that all strains clustered according to their assigned species, and node placements were supported by bootstrapping >80% (Fig. S1). This analysis determined that the seven phylogeny marker genes for *S. warneri* were identical for five of the six isolates and all strains formed a distinct clade. Similarly, two isolates of *S. pasteuri* could not be distinguished based on the analysis of the seven marker genes.

### Antimicrobial resistance

**Fig. 3.**
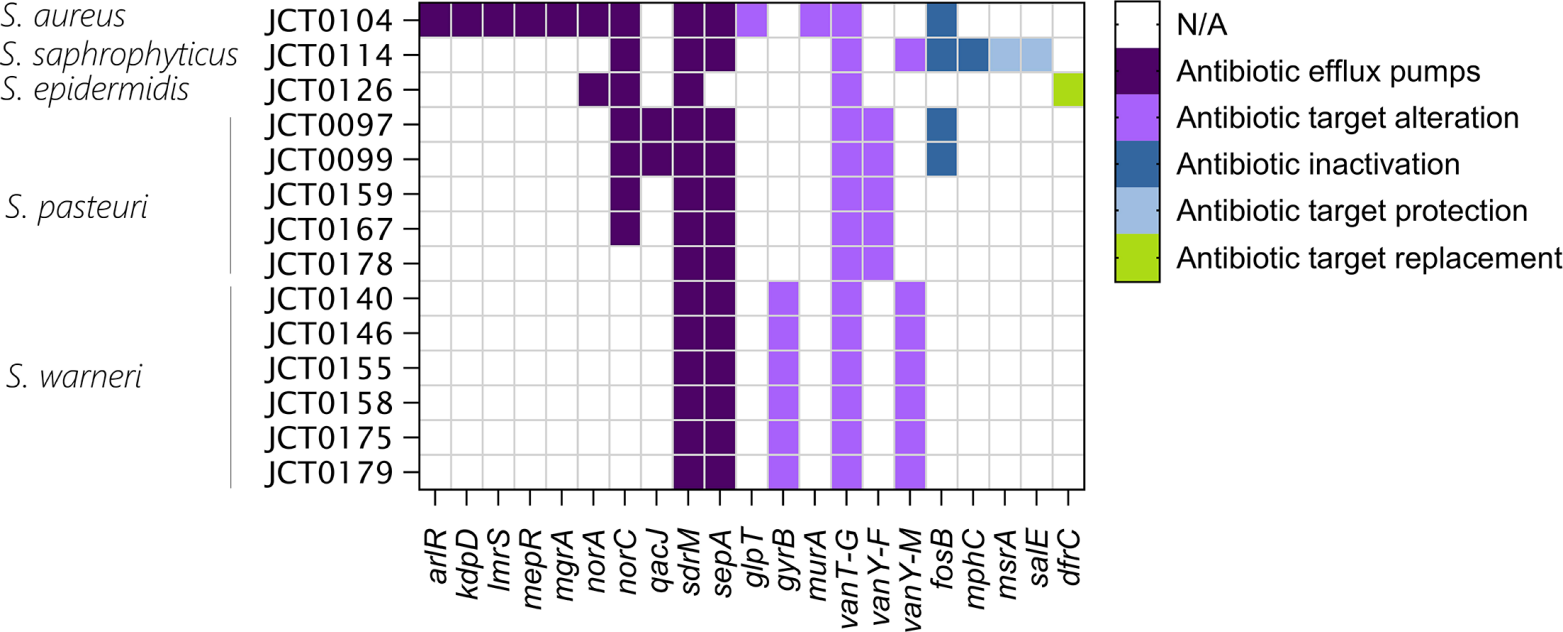
Heatmap displaying the distribution of AMR genes across staphylococcal strains colour coded by the mechanism of resistance.

Genomic AMR predictions using CARD identified a total of 85 AMR determinants, meeting either the strict or perfect cut-off set by CARD, across the 14 isolates. These determinants are associated with resistance to a range of antibiotics including beta-lactams, tetracyclines, macrolides, fluoroquinolones, glycopeptides, lincosamides, diaminopyrimidines, phosphonic acids, antiseptics, disinfectants and quaternary ammonium compounds (Fig. 3). The majority of the determinants identified (79/85) met the ‘strict’ cut-off (a strict match is not identical to the reference sequence, but the bit-score of the matched sequence is greater than the curated blastp bit-score cut-off). A total of six hits were identified as ‘perfect’, i.e. have 100% query identity and coverage with the reference sequence. These were *arlR*, *mepR* and *mgrA* in *S. aureus* JCT0104, *qacJ* in *S. pasteuri* JCT0097 and JCT0099 and *dfrC* in *S. epidermidis* JCT0126. The primary resistance mechanisms involved multidrug efflux pumps, target alteration and proteins that modulate gene expression for efflux pumps or cell wall biosynthesis enzymes. The isolate predicted to encode the highest number of AMR genes was *S. aureus* strain JCT0104 with 13. However, this strain does not carry either of the *mecA*/*mecC* genes for methicillin resistance, suggesting that it is most likely a methicillin-sensitive strain.

To confirm the resistances predicted by CARD, we tested all 14 isolates for phenotypic resistance by means of disc diffusion assays against a panel of 7 antibiotics [tetracycline, cefoxitin (beta-lactam), gentamycin (aminoglycoside), erythromycin (macrolide), sulfamethoxazole and trimethoprim (diaminopyrimidine), rifampicin and clindamycin (lincosamide); Table S3]. We observed a high degree of discordance between CARD predictions and our phenotypic findings, with only a single case of clinical resistance being identified; JCT0114 was resistant to erythromycin, presumably due to the presence of genes *mphC* and/or *msrA*. We noted numerous cases of discordance where bioinformatically predicted discrete resistance mechanisms did not engender phenotypic resistance. Examples include the identification of the dihydrofolate reductase gene, *dfrC*, in the genome of JCT0126 (a perfect hit), but the isolate was sensitive to trimethoprim and JCT0114 remaining sensitive to lincosamide despite the predicted presence of the ribosomal protection protein, SalE (strict hit).

### Virulence genes

VFanalyzer identified a total of 239 virulence genes across 14 isolates (Fig. 2), with enzymes comprising the majority at 33% (*n*=78). All 14 strains harboured *lip* and *nuc*, responsible for modifying host lipids and degrading nucleic acids, respectively. The complete *sspABC* operon, encompassing V8 protease (*sspA*), staphopain B (*sspB*) and staphylostatin B (*sspC*), was harboured by all isolates, except *S. epidermidis* strain JCT0126 and *S. saprophyticus* strain JCT0114; yet, *sspA* was present in all isolates and is predicted to digest host opsonins marking cells for phagocytosis. As expected, *S. aureus* encoded the most enzyme-related genes, with two key genes being *coa* and *sak*, which contribute to the production and dissolution of blood clots, respectively.

Immune evasion genes were the third most abundant group, comprising 16% (*n*=38) of all the identified virulence genes. However, there was no single immune evasion gene present across all strains. The most prevalent of these were the *capBCAD* operon genes, namely, *capB* and *capC* that contribute to the synthesis of an anti-phagocytic poly-gamma-glutamic acid layer. Additionally, among the adherence genes, two *S. pasteuri* strains carried *manA*, responsible for mannose-specific adherence to host tissues, while *S. aureus* exclusively harboured immune evasion genes *adsA*, *chp*, *snc* and *sbi*.

Toxins, constituting 8% (*n*=21) of virulence genes, were predominantly found in *S. aureus*. Among them were *set/sel* group genes associated with *Staphylococcus* enterotoxin production, commonly linked to food poisoning. Furthermore, the *S. aureus* strain carried genes for alpha (*hla*), delta (*hld*) and gamma (*hlg*-operon) haemolysin. In contrast, *S. epidermidis* exclusively possessed the *hlb* beta-haemolysin gene. Additionally, four strains of *S. pasteuri* and two strains of *S. warneri* harboured the *cylR* gene, responsible for pore-forming cytolysin production.

Genes associated with the type VII secretion system represented the fifth most abundant set, accounting for 8% (*n*=19) of the virulence genes. These genes were exclusively identified in isolates of *S. warneri* and *S. aureus*. Finally, iron uptake (*vctC*) accounted for 3% of all virulence genes identified (*n*=7) and was harboured by *S. warneri* and *S. saprophyticus*. Finally, serum resistance (*wbtE*) accounted for 3% of all virulence genes identified (*n*=6) and was present exclusively in S. *warneri*.

### Adherence

**Fig. 4.**
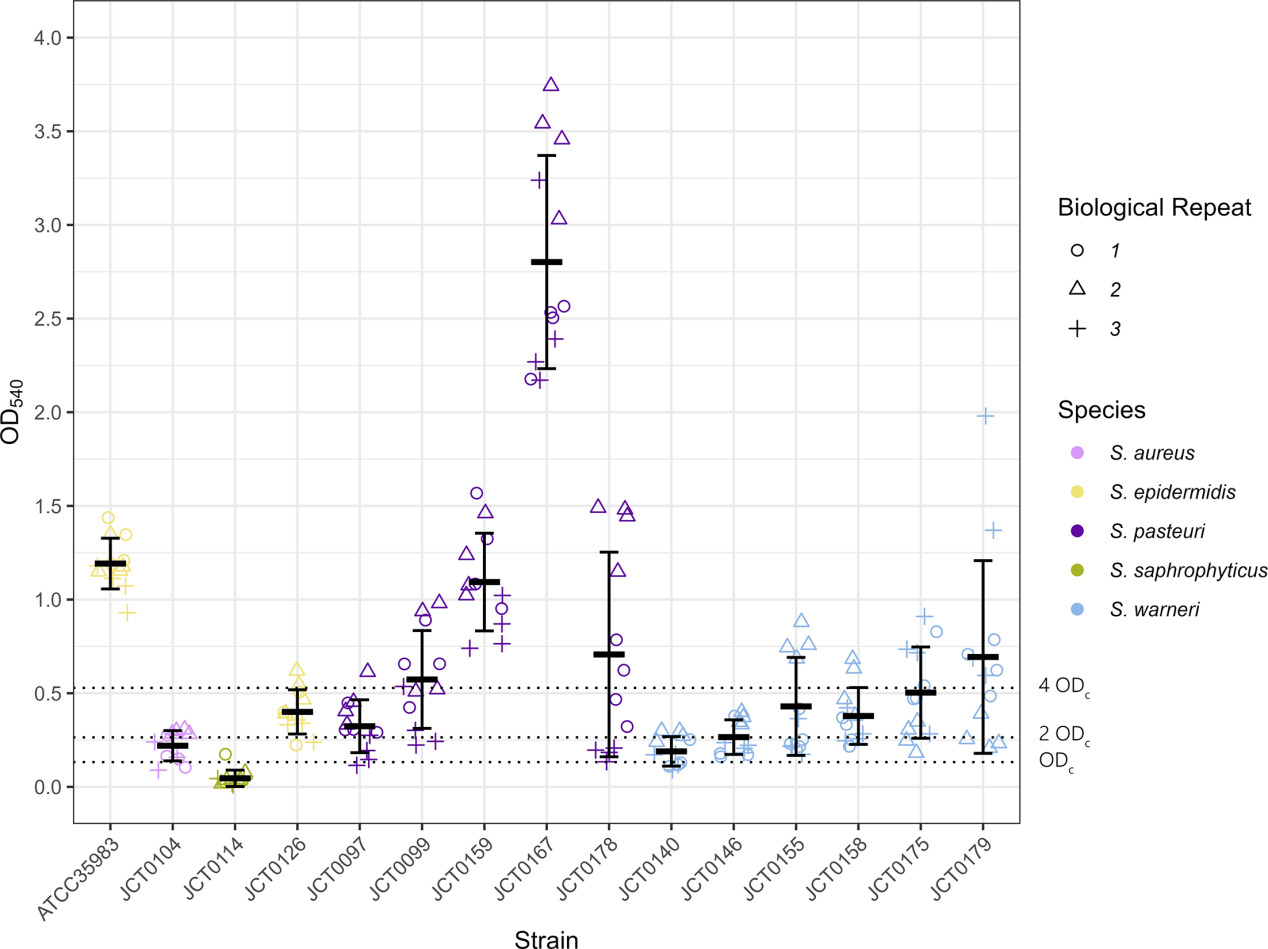
The biofilm-forming capabilities of staphylococcal strains. Each coloured point denotes an individual OD measurement (four technical and three biological replicates). Horizontal bars depict the average OD, and error bars represent the sd. Dotted lines represent boundaries for biofilm-forming categories. Non-adherent (OD_i_≤OD_C_), weakly adherent (OD_C_<OD_i_≤2 OD_C_), moderately adherent (2 OD_C_<OD_i_≤4 OD_C_) or strongly adherent (4 OD_C_<OD_i_).

Genomic analysis revealed that adherence genes were the second most represented group of virulence genes identified by VFanalyzer, totalling 70 associated genes across the 14 isolates (Fig. 2). All isolates formed either a weak, moderate or strong biofilm, except for *S. saprophyticus* isolate JCT0114, which was deemed to be non-adherent (Fig. 4 and raw data presented in Table S4).

*S. pasteuri* strains exhibited the greatest phenotypic adherence. This proficiency was supported by the presence of biofilm-associated adherence genes in all *S. pasteuri* strains, including polysaccharide intracellular adhesion (PIA) genes (*icaA*, *icaB* and *icaC*), extracellular matrix-binding protein (*ebp*) and autolysin (*atl*). Interestingly, *S. pasteuri* strains that produced comparatively weaker biofilms harboured additional adherence genes such as *sdrE*, *sdrD* and *eph*, known for their role in host tissue adherence.

*S. warneri* strains exhibited variable biofilm adherence capabilities and were categorized as weakly adherent, moderately adherent or strongly adherent. All *S. warneri* strains carried adherence genes *atl*, *ebp* and *sdrE* responsible for hydrolysing peptidoglycan, adhering to the extracellular matrix and adhering to host tissue, respectively. Notably, two strains (JCT0155 and JCT0158) harboured an additional adherence gene (*clfA*) responsible for host tissue adherence.

*S. aureus* strain JCT0104 harboured the most adhesion-related genes (*n*=13) but conversely formed a weakly adherent biofilm. Notably, JCT0104 was the only strain predicted to possess the *icaR* regulatory gene in addition to PIA genes *icaA*, *icaB* and *icaC* genes. *S. epidermidis* strain JCT0126 produced a moderate biofilm and carried three adherence genes, while *S. saprophyticus* strain JCT0114 formed a weakly adherent biofilm, exhibiting the lowest overall production, and harboured four adherence genes.

### Prediction of prophages

PHASTEST was used to detect the presence of intact prophages within the genomes of the 14 isolates. In total, 12 intact prophages were predicted across 11 isolates (Table 2). Intact prophages were confined to the genomes of isolates belonging to *S. warneri*, *S. pasteuri* and *S. aureus*. All the prophages detected within the genomes of the *S. warneri* isolates were found to be genetically very similar, sharing close to 100% ANI (Fig. S2). Despite their conserved nature, they appeared to share little genetic similarity with their closest identified bacteriophage relative, *Staphylococcus* prophage IME1354_01 (NC_070727.1) (Fig. S3C). Based on our ViPTree analysis, the closest relatives to the intact *S. pasteuri* prophages JCT0159_p1 and JCT0167_p1 were *Staphylococcus* phage vBSpsSQT1 and the closest relative to JCT0097_p1 and JCT0099_p1 was *Staphylococcus* phage IME-SA4, while the second prophage from isolate JCT0099 (JCT0099_p2) shared closest similarity to *Staphylococcus* phage IME_1348.

**Table 2.** Prophages predicted by PHASTEST and closest relative identified by VipTree by phylogenetic analysis of phage proteomes. The genomic similarity score (S_G_) in comparison to the closest predicted relative is presented in brackets as a value from 0 to 1

Species	Lab ID	Prophage ID	VipTree predicted closest relative
*S. pasteuri*	JCT0097	JCT0097_p1	*Staphylococcus* phage IME-SA4 (0.4406)
	JCT0099	JCT0099_p1	*Staphylococcus* phage IME-SA4 (0.6441)
	JCT0099	JCT0099_p2	*Staphylococcus* phage IME1348_01 (0.5485)
	JCT0159	JCT0159_p1	*Staphylococcus* phage vBSpsSQT1 (0.4513)
	JCT0167	JCT0167_p1	*Staphylococcus* phage vBSpsSQT1 (0.3881)
*S. warneri*	JCT0140	JCT0140_p1	*Staphylococcus* phage IME1354_01 (0.2361)
	JCT0146	JCT0146_p1	*Staphylococcus* phage IME1354_01 (0.2361)
	JCT0155	JCT0155_p1	*Staphylococcus* phage IME1354_01 (0.2345)
	JCT0158	JCT0158_p1	*Staphylococcus* phage IME1354_01 (0.2345)
	JCT0175	JCT0175_p1	*Staphylococcus* phage IME1354_01 (0.2361)
	JCT0179	JCT0179_p1	*Staphylococcus* phage IME1354_01 (0.2361)
*S. aureus*	JCT0104	JCT0104_p1	*Staphylococcus* phage JS01 (0.9013)

Genomic alignments again highlight that these prophages share little similarity with their closest known relatives (Fig. S3a, b) with the exception of *S. aureus* JCT0104_p1, which is highly similar to *Staphylococcus* phage JS01. No intact, questionable or incomplete prophages were found in the genomes of the *S. epidermidis* or *S. saprophyticus* isolates.

As lysogenic phage can transfer genetic material between bacterial strains, all intact prophages were screened for the presence of AMR and virulence genes. No CARD AMR predictions were made, but VFanalyzer predicted that JCT0104_p1 (*S. aureus*) prophage encoded staphylokinase (*sak*), which contributes to human-specific immune evasion [41].

## Discussion

Similar to humans, NHPs are predicted to carry numerous species of staphylococci in high abundance on their skin [42]. However, differences in diversity and distribution of staphylococci on the skin of humans and NHPs are expected due to the variation in the distribution of apocrine and eccrine sweat glands, differences in hygiene behaviours and host evolutionary history [42,44]. There are currently a limited number of studies describing the carriage and genetic diversity of staphylococci that colonize the skin of our closest primate relatives. This report represents the first investigation of the presence and characterization of staphylococci isolated from the skin of captive ring-tailed lemurs, black lemurs, spider monkeys, common marmosets and cotton-top tamarins.

### *Staphylococcus* spp. carriage by NHPs

The diversity of *Staphylococcus* spp. recovered from an individual animal is likely to be highly dependent on the physical site sampled and the sampling method employed. Our study included a range of NHPs housed at a UK zoo, which have frequent contact with humans during hand-feeding. We therefore sampled the palms of individual primates to assess the genetic diversity of staphylococci present and determine if potentially pathogenic isolates were present and so may pose a risk of zoonosis if transmission occurred. We recovered 14 presumptively unique staphylococcal isolates, based on differences in their 16S rRNA gene sequences and origins of isolation. These 14 isolates belonged to 5 different *Staphylococcus* spp. The majority of the isolates (93%, *n*=13/14) were CoNS with only a single isolate identified as *S. aureus*. Conversely, others have identified a diverse range of *S. aureus* strains isolated from both captive and wild NHPs [15,45,48]. However, these studies all sample from the nasal cavity, which in humans is known to be associated with higher carriage rates of *S. aureus* [49]. Most of these studies focused solely on the isolation of CoPS or more specifically *S. aureus* and did not attempt to characterize CoNS present. There are relatively few NHP studies reporting the full diversity of *Staphylococcus* spp. isolated from sites other than the nasal/oral cavity. However, it has previously been shown that the skin microbiota of captive saddle-back tamarins (*Saguinus fuscicollis*) is dominated by *Streptococcus* spp. and CoNS [50], including *S. haemolyticus*, *S. epidermidis*, *Staphylococcus simulans*, *S. warneri*, *S. saprophyticus* and *Staphylococcus sciuri*, which has been reclassified as *Mammaliicoccus sciuri* [51] (listed in order of prevalence). In humans, CoNS are regarded as ubiquitous colonizers of the skin, whereas *S. aureus* is considered a relatively poor skin colonizer and is more frequently associated with the anterior nares [52,54]. Our results, in agreement with those of Nordstrom *et al*., suggest that like in humans, CoNS, rather than CoPS, are the major colonizer of NHP skin.

### NHP species-specific findings

The four NHP species investigated in this study fall into distinct groupings based on their evolutionary history and characteristics. Spider monkeys, common marmosets and cotton-top tamarins are all classified as New World monkeys that are closely related to human primates and Old-World monkeys. In contrast, lemurs belong to the prosimian group of primates and are considered to have diverged earlier in the evolution of the order.

Lemurs (ring-tailed and black) exhibited the highest prevalence of *Staphylococcus* among the four NHP species, often harbouring multiple strains of *S. warneri* and *S. pasteuri*. Notably, *S. warneri* and *S. pasteuri* strains isolated from individual lemurs were genetically very similar, sharing >99% FastANI scores for strains within each species, with little variation in terms of predicted virulence factors and antimicrobial determinants. There are no prior reports of * S. pasteuri* colonizing the skin of lemurs, although *S. warneri* has been isolated from the eyelid of a ring-tailed lemur [55]. Additional reports of *Staphylococcus* colonization in lemurids are limited to *S. aureus*, identified in the oropharyngeal, rectal, vaginal, nasal and mucosal regions [56,57]. The evolutionary divergence between humans and lemurs is substantial and likely contributes to differences in the skin microbiome. Both *S. warneri* and *S. pasteuri* have been recovered from healthy human skin, but *S. warneri* is known to be an infrequent colonizer [4]. Spider monkeys share a closer genetic relationship with humans compared to lemurids; in this study, spider monkeys displayed the most diverse *Staphylococcus* spp. carriage with *S. aureus*, *S. pasteuri* and *S. saprophyticus* recovered from swabs. Currently, there are no other reports documenting the recovery of *Staphylococcus* spp. from spider monkeys. Common marmosets exhibited the lowest abundance and diversity of staphylococci, with only *S. epidermidis* isolated from a single primate. No isolates were recovered from cotton-top tamarin swabs.

### ST and population structure

Owing to their contribution to human disease, *S. epidermidis* and *S. aureus* are two of the most extensively studied *Staphylococcus* species. Therefore, we were able to further investigate their ST and clonality as MLST public databases are available [38,39]. *S. epidermidis* strain JCT0126 was found to belong to ST621 and GC 5. Strains belonging to GC5 are common among clinical samples and are generally enriched for hospital-associated markers including antibiotic resistance and high biofilm production; however, JCT0126 was susceptible to all tested antibiotics and was a moderate biofilm producer. MSSA *S. aureus* strain JCT0114 was part of ST39 and a predicted member of CC30. CC30 is a human lineage commonly found residing in the nasal passage and is notorious for causing human infections and is associated with the epidemic MRSA-16 strain (EMRSA-16) [58,59]. Interestingly, MRSA and MSSA CC30 strains have been recovered from livestock, wild animals and companion animals worldwide. A few examples include pigs in Northern Ireland, Portugal, Poland and Denmark, goats in Nigeria and Italy, cows in Portugal, ribbon fish in India, camels in Kenya, buzzard in Portugal and cats and dogs in Tunisia [60,70]. Many of these strains were found to carry virulence and AMR determinants. In particular, two MRSA ST39 CC30 strains isolated from pigs in Hong Kong harboured enterotoxins, toxic shock syndrome toxin, staphylokinase and human immune evasion clusters, with the latter suggesting this strain could cause human infections [71]. In contrast, *S. aureus* JCT0104 (ST39, CC30) isolated from a spider monkey lacked many of these important toxins. Previously, an ST39 or CC30 strain has not been recovered from an NHP host; however, an ST30, CC30 *S. aureus* strain positive for PVL toxin and penicillin resistance has been recovered from the skin of a human sanctuary worker who had close interactions with chimpanzees [72].

### Antimicrobial resistance

The worsening pandemic of antibiotic resistance poses a global health challenge that threatens the efficacy of treatment for bacterial infections. Encouragingly, we found limited evidence of AMR associated with staphylococci isolated in our study despite CARD predicting multiple resistances against a range of antibiotic classes. Others have also described the issue of genotype–phenotype discordance with regard to AMR predictions made bioinformatically using WGS sequence data for the genus *Staphylococcus* [73]. There are a number of possible explanations for this apparent discordance. Firstly, a number of the resistance determinants identified by CARD are not ‘resistance genes’ *sensu stricto*. For example, *mgrA*, *arlR* and *kdpD* are regulatory elements, which have been proposed to be involved in resistance mechanisms but do not encode specific resistance determinants per se [74,76]. Secondly, the majority of the determinants were strict hits, meaning they were not identical to the database reference sequences. Such hits may include partial or mutated gene sequences, which no longer encode functional resistance mechanisms. Lastly, a number of the identified resistance determinants are associated with non-specific efflux pumps (e.g. *LmrS*, *norA*, *norC*, *qacJ*, *sdrM* and *sepA*) and their expression levels and contribution to resistance to any one antibiotic can be difficult to quantify. Moreover, the efflux pumps encoded by *qacJ* and *sepA* have been identified as providing resistance against disinfectants and antimicrobial dyes rather than specific antibiotics [77,78].

We identified only a single case of clinical resistance (*S. saprophyticus* strain JCT0114, resistant to erythromycin) when we screened our collection against a panel of seven antibiotics belonging to a range of drug classes. The literature consistently highlights minimal antibiotic resistance levels among NHP-derived *Staphylococcus* spp. [45,46, 79], especially in CoNS. For example, a previous study describing the isolation of 62 staphylococcal strains (CoNS and CoPS) from the nasal cavities of NHPs reported only a single case of intermediate resistance when the strains were tested against a panel of 15 antimicrobial agents covering a wide range of different classes [46]. Similarly, Schaumburg *et al*. isolated 58 *S*. *aureus* strains from African NHPs and found only a single case of penicillin resistance [45]. Moreover, in instances where elevated antibiotic resistance rates were observed in staphylococcal strains derived from NHPs, there were reports of prior antibiotic treatments or potential transmission of resistant strains from human sanctuary workers to NHPs [72].

### Adherence

Staphylococci enclosed within a biofilm are deemed more difficult to treat owing to their decreased sensitivity to antibiotics and host immune defences. To our knowledge, this study marks the first report on the phenotypic biofilm-forming capabilities of staphylococcal strains derived from NHPs. We observed that all strains formed biofilm to some degree with the exception of JCT0114. The most proficient biofilm-forming strains exclusively harboured crucial biofilm-associated genes, such as the *ica* operon. *S. aureus* strain JCT0104 harboured the most adhesion-related genes (*n*=13) but conversely formed a weakly adherent biofilm. Notably, JCT0104 was the only strain predicted to possess the *icaR* regulatory gene in addition to PIA genes *icaA*, *icaB* and *icaC* genes. As *icaR* is a negative regulator of the *ica* operon, the presence of *icaR* in the genome of JCT0104 may explain why our *S. aureus* strain produced a weak biofilm compared to the strong biofilms formed by the *S. pasteuri* isolates (which harbour the *ica* operon but lack the negative regulator). Others have noted that a deletion or inactivation of *icaR* results in increased expression of the *ica* operon and concomitant increase in biofilm production [80,81]

Hoefer *et al*. previously screened *S. epidermidis* strains isolated from vervet monkeys for the presence of biofilm-associated genes (*ica*RADBC operon, *embp*, *aap*, *bhp* and IS*256*) but identified none [82]. Others have also reported a low prevalence of biofilm-associated genes among isolates derived from animals. For example, Argudin *et al*. compared the prevalence of the *ica* operon between *S. epidermidis* strains isolated from animals and humans and found a relatively low number of animal isolates encoded these genes (16.5% prevalence in animal isolates, *n*=103, vs 36%, *n*=160, in human isolates) [83]. Thus, our study appears to contradict the broader trend in the literature, which reports low levels of adherence associated with staphylococci isolated from animals.

### Prophage

As prophages may act as mediators of genetic transfer between bacterial populations [84,85], identifying their presence and associated AMR or virulence determinants may provide insights into how these genes are transferred between strains. We identified a total of 12 intact prophages (with a mean genome size of 41,718 bp) encoded in the genomes of 11 of our isolates. We note that N50 values achieved for some of our assemblies were lower than the mean prophage genome size, and this may have hindered the identification of intact prophages within some of the host genomes. For example, prophages JCT0099_p1 and JCT0099_p2 span the entire length of their respective contigs and appear to be missing a number of regulatory elements. It is highly likely that these sequences are truncated and were incorrectly identified as ‘intact’ by PHASTEST. A single potential virulence gene was identified, *sak*, belonging to prophage JCT0104_p1 encoded by the genome of our single *S. aureus* isolate. Staphylokinase is known to be bacteriophage encoded and plays an important role in virulence through mediating resistance to host antimicrobial peptides and via the formation of plasmin, a proteolytic enzyme which facilitates tissue damage [86]. We also observed that prophages contribute to the genetic diversity of closely related strains. For example, isolates JCT0097 and JCT0159 share 99% ANI but were predicted to encode differing intact prophages.

## Conclusion

Although our sample size was relatively small, this study contributes to our limited knowledge on the diversity of staphylococci residing on the skin of captive NHPs. Our results highlight that CoNS are likely to be the dominant group of staphylococci found on NHP skin. We also report, like others, that staphylococci residing on NHPs have limited antibiotic resistance.

## supplementary material

10.1099/mic.0.001546 Uncited Supplementary Material 1.

10.1099/mic.0.001546 Uncited Supplementary Material 2.

## Abbreviations

AMR: antimicrobial resistance
ANI: average nt identity
CARD: comprehensive antibiotic resistance database
CC: clonal complex
CoNS: coagulase-negative staphylococci
CoPS: coagulase-positive staphylococci
EUCAST: European Committee on Antimicrobial Susceptibility Testing
GC: genetic cluster
MRSA: methicillin-resistant *Staphylococcus aureus*
MSA: mannitol salt agar
MSSA: methicillin-susceptible *Staphylococcus aureus*
NHP: non-human primate
PIA: polysaccharide intracellular adhesion
rMLST: ribosomal MLST
ST: sequence type
TSB: tryptone soy broth
WGS: whole-genome sequencing
