# Supplementary material for: Genomic and phenotypic characterization of staphylococci isolated from the skin of non-human primates
Source: Microbiology (Reading). 2025 Mar 25;171(3):001546. doi: 10.1099/mic.0.001546 (PMC11936348; doi:10.1099/mic.0.001546)
Supplement: Uncited Supplementary Material 1. [file mic-171-01546-s001.pdf]

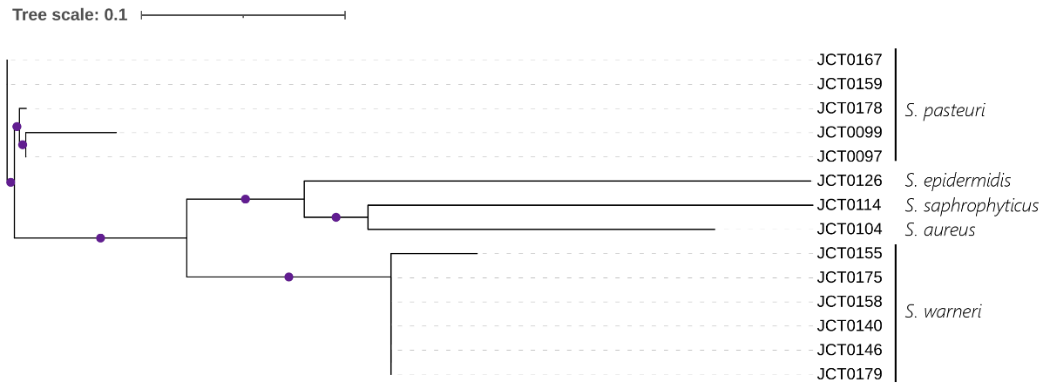

Supplementary Figure 1: Phylogenetic analysis of the *Staphylococcus* isolates. Maximum likelihood tree generated by concatenating the nucleotide sequences of 7 orthologous group genes (*mutS, recD2, pepA, yheS, pheT, pbp3* and *rpoC*) and aligning the concatenated sequences across the 14 strains, as proposed by Graña-Miraglia et al., 2018. Bootstraps are presented as a percentage of 1000 replicates, values over 80% are displayed as purple circles in the centre of branches. Scale bar refers to the nucleotide substitutions per site.

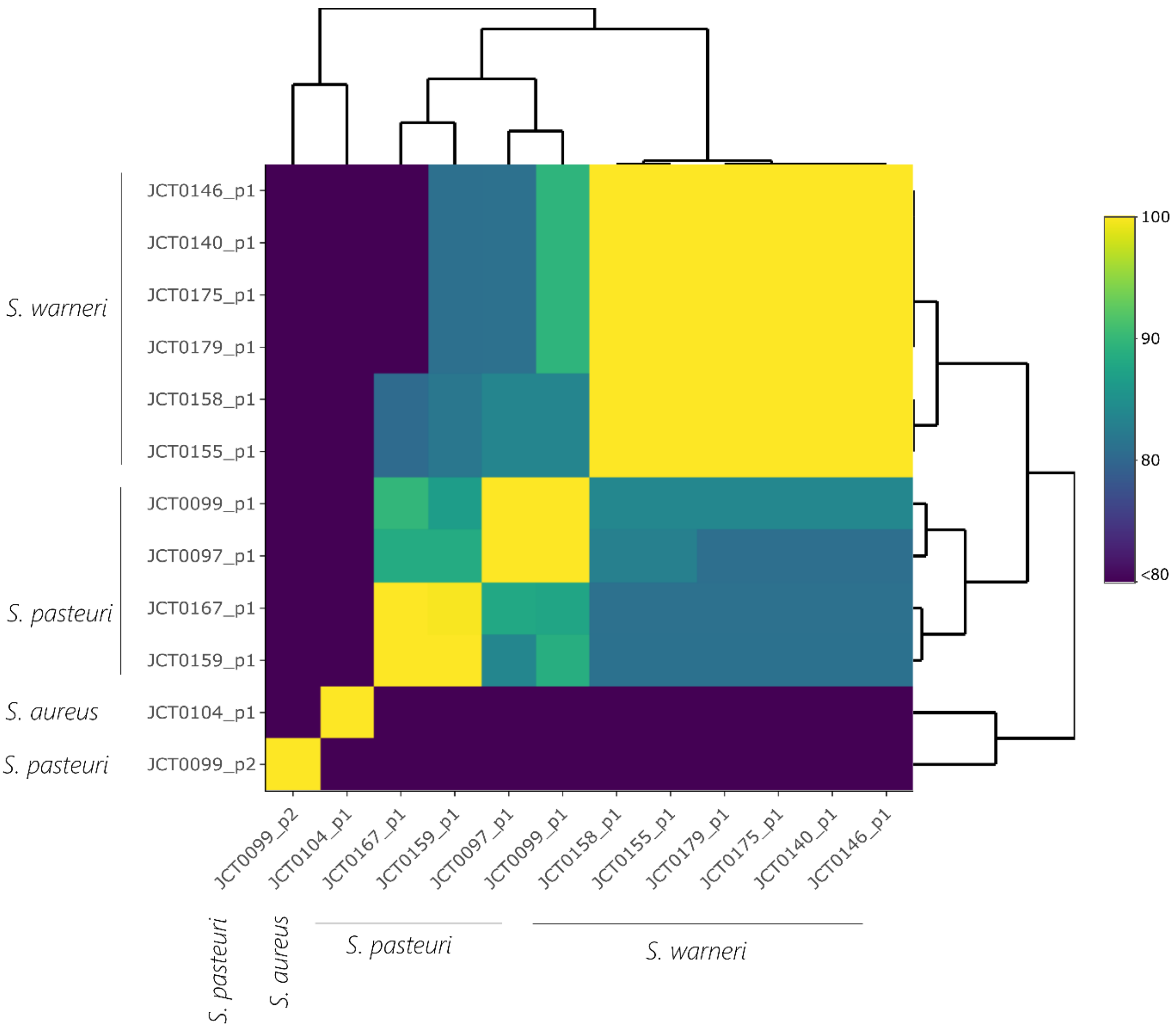

Supplementary Figure 2: Dendrogram and heatmap of hierarchical clustering of average nucleotide identity (ANI) between 12 *Staphylococcus* JCT prophages (query and references) using FastANI. Where FastANI could not calculate a score due to similarity falling below the threshold, a value <80 has been presented.

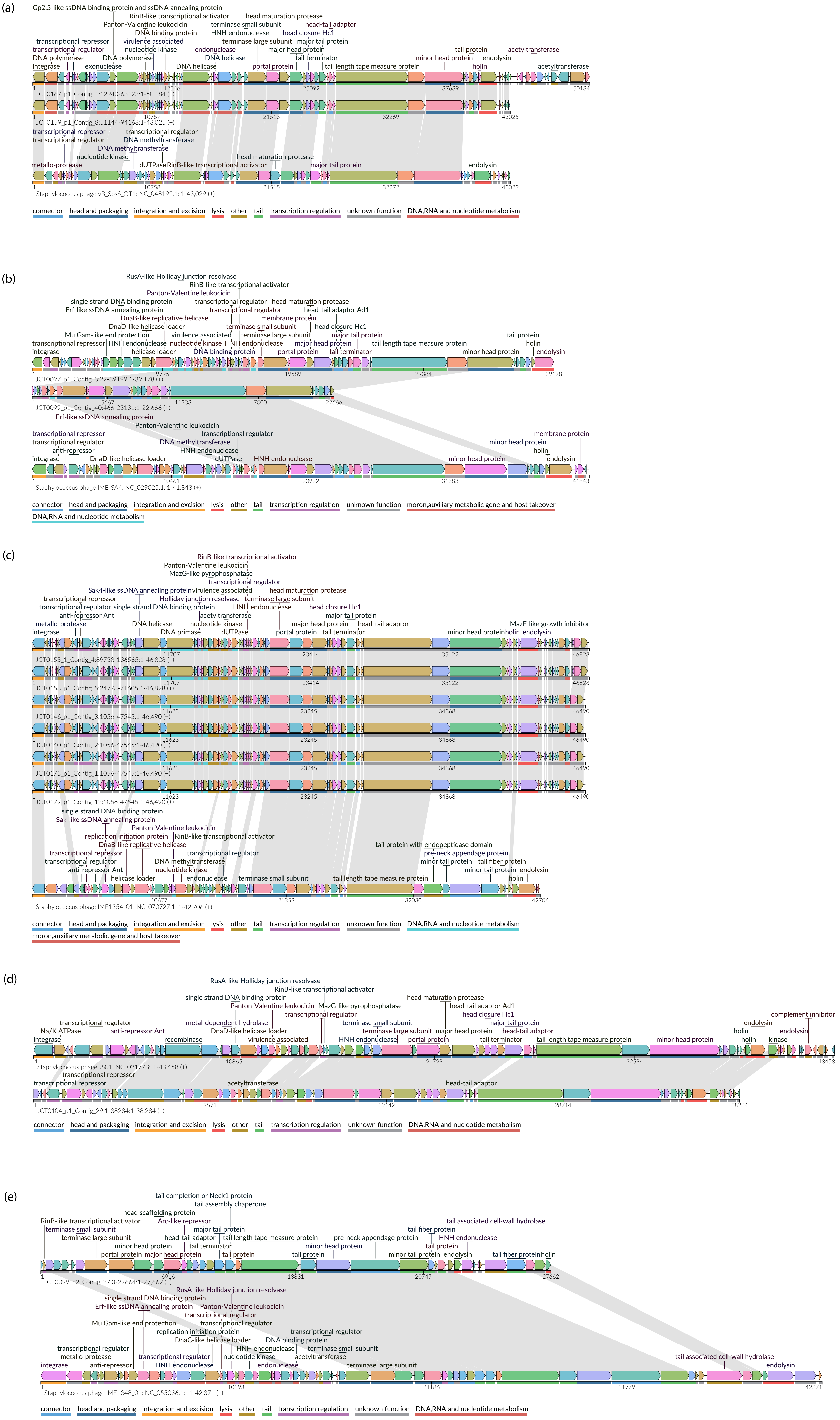

(a)
Gp2.5-like ssDNA binding protein and ssDNA annealing protein
RinB-like transcriptional activator
Panton-Valentine leukocicin
DNA binding protein
transcriptional repressor
transcriptional regulator
DNA polymerase
integrase
virulence associated
nucleotide kinase
DNA polymerase
exonuclease
endonuclease
DNA helicase
DNA helicase
terminase small subunit
HNH endonuclease
terminase large subunit
head maturation protease
head-tail adaptor
head closure Hc1
major tail protein
major head protein
tail terminator
portal protein
tail length tape measure protein
tail protein
minor head protein
endolysin
holin
acetyltransferase
acetyltransferase
JCT0167_p1_Contig_1:12940-63123:1-50,184 (+)
JCT0159_p1_Contig_8:51144-94168:1-43,025 (+)
transcriptional repressor
transcriptional regulator
transcriptional regulator
DNA methyltransferase
DNA methyltransferase
nucleotide kinase
metallo-protease
dUTPase
RinB-like transcriptional activator
head maturation protease
major tail protein
endolysin
Staphylococcus phage vB_SpsS_QT1: NC_048192.1: 1-43,029 (+)
connector
head and packaging
integration and excision
lysis
other
tail
transcription regulation
unknown function
DNA,RNA and nucleotide metabolism
(b)
RusA-like Holliday junction resolvase
RinB-like transcriptional activator
Panton-Valentine leukocicin
single strand DNA binding protein
Erf-like ssDNA annealing protein
transcriptional regulator
transcriptional regulator
head maturation protease
head-tail adaptor Ad1
DnaB-like replicative helicase
membrane protein
DnaD-like helicase loader
terminase small subunit
head closure Hc1
Mu Gam-like end protection
virulence associated
terminase large subunit
major tail protein
transcriptional repressor
HNH endonuclease
nucleotide kinase
HNH endonuclease
major head protein
tail length tape measure protein
tail protein
holin
integrase
helicase loader
DNA binding protein
portal protein
tail terminator
minor head protein
endolysin
JCT0097_p1_Contig_8:22-39199:1-39,178 (+)
JCT0099_p1_Contig_40:466-23131:1-22,666 (+)
Erf-like ssDNA annealing protein
Panton-Valentine leukocicin
transcriptional repressor
transcriptional regulator
transcriptional regulator
DNA methyltransferase
HNH endonuclease
anti-repressor
integrase
DnaD-like helicase loader
dUTPase
HNH endonuclease
membrane protein
minor head protein
holin
minor head protein
endolysin
Staphylococcus phage IME-SA4: NC_029025.1: 1-41,843 (+)
connector
head and packaging
integration and excision
lysis
other
tail
transcription regulation
unknown function
moron,auxiliary metabolic gene and host takeover
DNA,RNA and nucleotide metabolism
(c)
RinB-like transcriptional activator
Panton-Valentine leukocicin
MazG-like pyrophosphatase
transcriptional regulator
Sak4-like ssDNA annealing protein
virulence associated
head maturation protease
transcriptional repressor
Holliday junction resolvase
terminase large subunit
transcriptional regulator
single strand DNA binding protein
HNH endonuclease
head closure Hc1
anti-repressor Ant
acetyltransferase
major tail protein
metallo-protease
DNA helicase
nucleotide kinase
major head protein
head-tail adaptor
MazF-like growth inhibitor
integrase
DNA primase
dUTPase
portal protein
tail terminator
minor head protein
holin
endolysin
JCT0155_1_Contig_4:89738-136565:1-46,828 (+)
JCT0158_p1_Contig_5:24778-71605:1-46,828 (+)
JCT0146_p1_Contig_3:1056-47545:1-46,490 (+)
JCT0140_p1_Contig_2:1056-47545:1-46,490 (+)
JCT0175_p1_Contig_1:1056-47545:1-46,490 (+)
JCT0179_p1_Contig_12:1056-47545:1-46,490 (+)
single strand DNA binding protein
Sak-like ssDNA annealing protein
Panton-Valentine leukocicin
replication initiation protein
RinB-like transcriptional activator
DnaB-like replicative helicase
tail protein with endopeptidase domain
transcriptional repressor
transcriptional regulator
pre-neck appendage protein
transcriptional regulator
DNA methyltransferase
minor tail protein
tail fiber protein
anti-repressor Ant
nucleotide kinase
minor tail protein
endolysin
helicase loader
endonuclease
terminase small subunit
tail length tape measure protein
holin
Staphylococcus phage IME1354_01: NC_070727.1: 1-42,706 (+)
connector
head and packaging
integration and excision
lysis
other
tail
transcription regulation
unknown function
DNA,RNA and nucleotide metabolism
moron,auxiliary metabolic gene and host takeover
(d)
RusA-like Holliday junction resolvase
RinB-like transcriptional activator
single strand DNA binding protein
head maturation protease
Panton-Valentine leukocicin
MazG-like pyrophosphatase
head-tail adaptor Ad1
transcriptional regulator
head closure Hc1
complement inhibitor
transcriptional regulator
metal-dependent hydrolase
terminase small subunit
major tail protein
endolysin
Na/K ATPase
anti-repressor Ant
DnaD-like helicase loader
terminase large subunit
major head protein
head-tail adaptor
holin
endolysin
integrase
recombinase
virulence associated
HNH endonuclease
portal protein
tail terminator
tail length tape measure protein
minor head protein
holin
kinase
Staphylococcus phage JS01: NC_021773: 1-43,458 (+)
transcriptional regulator
transcriptional repressor
acetyltransferase
head-tail adaptor
JCT0104_p1_Contig_29:1-38284:1-38,284 (+)
connector
head and packaging
integration and excision
lysis
other
tail
transcription regulation
unknown function
DNA,RNA and nucleotide metabolism
(e)
tail completion or Neck1 protein
tail assembly chaperone
head scaffolding protein
RinB-like transcriptional activator
Arc-like repressor
tail associated cell-wall hydrolase
terminase small subunit
major tail protein
tail fiber protein
terminase large subunit
head-tail adaptor
tail length tape measure protein
pre-neck appendage protein
HNH endonuclease
minor head protein
tail terminator
minor head protein
tail protein
portal protein
major head protein
tail protein
tail protein
minor tail protein
endolysin
tail fiber protein
holin
JCT0099_p2_Contig_27:3-27664:1-27,662 (+)
RusA-like Holliday junction resolvase
single strand DNA binding protein
Erf-like ssDNA annealing protein
Panton-Valentine leukocicin
transcriptional regulator
Mu Gam-like end protection
transcriptional regulator
replication initiation protein
transcriptional regulator
DnaC-like helicase loader
DNA binding protein
transcriptional regulator
HNH endonuclease
terminase small subunit
metallo-protease
transcriptional regulator
nucleotide kinase
acetyltransferase
tail associated cell-wall hydrolase
integrase
anti-repressor
HNH endonuclease
endonuclease
terminase large subunit
endolysin
Staphylococcus phage IME1348_01: NC_055036.1: 1-42,371 (+)
connector
head and packaging
integration and excision
lysis
other
tail
transcription regulation
unknown function
DNA,RNA and nucleotide metabolism

Supplementary Figure 3: Gene cluster comparisons between *Staphylococcus* JCT prophages and ViPTree-predicted closest phage relatives visualised using LoVis4u. Genomes have been reorientated to start with the integrase gene when present. The colour of arrows indicates homologous genes with connecting lines in grey further indicating regions of homology. Predicted phage modules are indicated by solid-coloured lines under corresponding regions of each genome.

(a) Comparison of JCT0159_p1 and JCT0167_p1 with the closest relative *Staphylococcus* phage vBSpsSQT1. (b) Comparison of JCT0099_p1 and JCT0097_p1 with the closest relative *Staphylococcus* phage IME-SA4. (c) Comparison of JCT0158_p1, JCT0155_p1, JCT0140_p1, JCT0179_p1, JCT0175_p1, and JCT0146_p1 with the closest relative *Staphylococcus* virus IME1354_01. (d) Comparison of JCT0104_p1 with the closest relative *Staphylococcus* phage JS01. (e) Comparison of JCT0099_p2 with the closest relative *Staphylococcus* phage IME-1348.
